# Our Correspondence

**Published:** 1869-04

**Authors:** 


### Our Correspondence.

DYER STATION, Tenn , March, 7th 1869.

T. S. UP DE GRAFF, M. D.,

*Dear Sir :*—Can you give me a specific—or anything approximating it—for Chronic Cystirrhœa? I have it myself—have had it occasionally for several years. It is a very unpleasant affliction. I have almost exhausted the Materia Medica, but received but temporary benefit. Would be a thousand times obliged to you for anything beneficial.

Ever Yours, Fraternally,
"——, M. D."

ANSWER.—Carbolic acid is the remedy, Doctor. Inject the bladder with warm, distilled water, containing the sol. of Carbolic Acid—about five drops to the pint, to commence with. Make this application twice a week, adding to its strength each time, being careful not to get it strong enough to cause smarting. We have seen good results from this treatment or would not recommend it to you.

---

HARRISBURG, Linn Co., Oregon, Nov. 29, 1868.

*Dear Sir:*—I chanced to see a number of your paper (the BISTOURY) and see that you answer through the columns of your paper those who write to you inquiring for medical aid, and take the liberty of addressing you.

About three years ago I was taken with a white swelling in my hip joint, and although it has got well, my hip joint is stiff so that I cannot use my leg, and I want to know the best remedy for a stiff joint.

If you will answer this through the columns of your paper and send me the number that contains the reply, you will confer a great favor upon me.

Yours, &c., J. B. R.

ANSWER:—You have *Intra-articular Anchylosis*, in which the *synovial*, or lubricating membrane of the joint is destroyed, through inflammation. In such cases too, a deposit of plastic matter accrues upon the surface of the bone causing it to adhere firmly to the bony socket.

There is no help for such cases. The more you meddle with it, the worse you will be off. Let it alone.

---

LEON, Alabama, Jan. 12th, 1869.

THAD. S. UP DE GRAFF, M. D.,

*Dear Sir :*—Your "BISTOURY" accidently fell into my hands a short time ago and I have been thinking that you could prescribe for my disease. I have had the "chills and fever" for two years, and all the Doctors in this country have failed to cure me. They have got all my money and no cure. They gave quinine, arsenic, iron, turpentine, pepper and whisky, and they have all failed. If you know of any remedy I would be more than thankful if you would send it to me. Direct to G. N.

ANSWER:—Evidently the best remedy for your disease, is to change your place of residence.—You must remove from the malarious district in which you now have your abiding place. Any case of intermittent fever that the proper use of quinine will not avert, requires a change of base. The sooner you leave, the better for you.

---

PARKVILLE, Mo., Feb. 25, 1869.

DR. UP DE GRAFF,

*Sir :*—Being a constant reader of your valuable Medical Journal, and noticing your kindness in furnishing recipes for different diseases, I write to you for a recipe in my case.

I have been afflicted with a sore nose for a year. The sores are confined to the nostrils.—Sometimes they come as little boils in the nostrils, again general soreness in each nostril. I have tried different ointments without effecting a permanent cure. You could give me the recipe in your paper. By so doing you will much oblige Yours, &c.,
W.

ANSWER:—Procure the following mixture:—

℞.

Pot. Iod: gr. x.

Com. Syr. Sarsap: ℥ vi.

Mix.

Take a tea-spoon-full morning and evening. After it has *all* been taken, apply this ointment to the sore—if it still remains—every night:

℞

Simple Cerate, ℥ i.

Carbolic Acid Sol. gtt. iv.

Fiat ung:

---

RUSHFORD, N. Y., March 11, 1869.

DR. T. S. UP DE GRAFF,

*Dear Sir :*

Several years ago I had inflammation of the eyes quite severely. I tried almost every remedy recommended—and almost every one had a remedy—and probably thereby aggravated the case. But after about eight weeks the inflammation subsided; but my eyes were left weak, and continue to be somewhat weak and somewhat inflamed occasionally. The lids are enlarged so that they do not open much more than half as wide as formerly. In the morning the balls are painful and red, especially after using them to read the previous evening.

Now what course would you recommend for strengthening them, and what, if anything can be done for the lids? Please answer in the BISTOURY, which has just come under my notice and with which I am highly pleased.

Yours Respectfully.
J. W.

ANSWER:—It is probable that you have a granular lid. If so, nothing but a thorough treatment under some competent Oculist, will restore your eyes to healthfulness. The best remedy to use yourself, is to bathe the eyes frequently in cold, salt water, and to take an occasional saline cathartic. This will give you temporary relief.

---

FRANKLINVILLE, Catt. Co., N. Y.,
January 31, 1869.

DEAR SIR: I wish to ask your advice in regard to my hair. It commenced falling off about four years ago, which was when I was eighteen years of age. The hair on the sides of my head is as thick as ever it was, but on the top it is very short and fine, and does not seem to grow any. It does not fall off any now. I have never been sick; but, on the contrary, have always been healthy and tough. So that cannot be the cause. I think it is owing to excessive perspiration. I wish to know if there is anything that can be done to promote a more vigorous growth, or if any of the preparations so highly recommended by their proprietors are good for anything. If it will not be encroaching too much upon your time, I shall be very glad to have you answer me by letter; but if you cannot, please do so through the columns of the BISTOURY. L. E.

ANSWER:—Dissolve one drachm of *pearlash* in one quart of soft water. Wet the scalp thoroughly with the solution, and rub it well with a towel. Rinse the hair in pure water, dry it well, and then apply bay rum with a stiff brush, rubbing it well into the scalp. This will cause considerable smarting for a moment, but the pearlash will dissolve all the sebacious matter deposited in the follicle of the hair, while the rum and friction will stimulate the hair to grow. This is the very best "hair invigorator" that can be employed. Those patent preparations for the hair contain preparations of lead, which is often dangerous to use.

---

HILLSBORO, N. C., 24th Feb. 1869.

DR. T. S. UP DE GRAFF:

*Dear Sir:*—I have delayed writing in reply to yours of the 17th June, in hopes of being able to get some information that would enable me to write more satisfactorily as to your proposed change in the BISTOURY. I have not for various reasons been able to do so. I am not prepared to say what your southern subscribers will do in this matter, but for myself I can say that I am pleased with the contemplated change, and it seems to me that others must be also. I think that a majority of those who have been getting the paper *free*, certainly cannot and will not hesitate to pay the small sum of thirty or fifty cents for it in its new dress.

Allow me to make a suggestion or two.—I think that a column of Domestic or Household Receipts would add to its interest, to the general reader. I further suggest that you put the subscription at fifty cents, as I think you will get about as many subscribers at fifty as you will at thirty, and you can then offer us a better paper. If you decide to make the change get out your April No. as early as you can. In conclusion, I promise to interest myself in behalf of the BISTOURY, and wish it great success,

Respectfully, J. S. L.

REPLY.—Much obliged to J. S. L. for his offer to interest himself in behalf of the BISTOURY. Hope he will be able to send us a large Club in his neighborhood, and we promise to make it the best investment in a journal our subscribers have ever made.

Hope that all our old subscribers in the south will continue their subscriptions now that we *charge* for the journal. But if any of them are unable to pay us fifty cents per year for the BISTOURY, if they will write us informing us of the fact, it shall be supplied to them *free*, as heretofore.

---

FORT MADISON, Lee Co., Iowa,
Jan. 15, 1869.

THAD S. UPDEGRAFF, M. D.

*Sir:*—Thanks for your valuable paper (the BISTOURY) which comes to hand regularly to my daughter—I have concluded to tax your patience with a communication, and fear its prolixity will tire you.

I am an old man of 74 years. Ten years since I had a high fever which left me with noises in the head and impaired hearing. I took no medicine to cure the fever but some pills. In the first place I tried tepid bathing and a tepid head bath, with much friction on the head. I still follow the plow, use no ardent spirits or tobacco. The above treatment restored my hearing some, but it is now getting worse. Seeing Dr. Stillwell's advertisement in the N. Y. papers, I wrote to him. He says he has been to London and Paris, and is better qualified than most of our physicians. Will guarantee a cure for $35, if not cured will doctor afterwards gratis. His "Organic Vibrator" to wear in the ears costs $10, and will enable the wearer to hear whilst used.

At this time I received a letter from an unknown lady of Blooklyn, N. Y. She says "prompted by christian charity, hearing of your affliction from a gentleman of our section, I enclose a slip from a newspaper, giving an account of an extraordinary cure of 15 years standing of deafness, which is certified to by an Episcopal Divine of N. Y.," and directs me to write to Mrs. Mary C. Leggett of Hoboken, N. J.—Mrs. Leggett writes that she is a widow of an officer in the army, and being deaf from scrofula with noises in the head, was placed by her husband under the care of a great Indian Doctor, who eradicated the scrofula and restored her hearing in five weeks.

Here follows her prescription given gratis:

"RECEIPT FOR MAKING THE INDIAN DEAFNESS REMEDY.—Oil of Dippel, 1 dram; Oil of Scorpion, half a dram; Oil of Charborte, half a dram; Seed of Prairie Wort, two ounces. Mix the oils together. Then get the oil from the Prarie Wort by expression. Mix with the other oils, and syringe the ears every night before retiring."

The oil penetrates through all obstructions. The effect is almost magical. Will send the medicine properly prepared by mail for five dollars, *for no other cause only to show her gratitude to God for her cure, and to relieve suffering humanity.*" Her receipt for scrofula I need not insert. I know your opinion of Indian doctors.

Please give me your advice. I would willingly pay for a cure, but not to be humbugged.

Enclosed is a stamp to prepay postage, or you can answer in the April number of the BISTOURY. Respectfully,

DANIEL MCCREADY.

ANSWER:—We have taken the liberty to publish Mr. McCready's letter entire, for the reason that it exposes in few words one very prevalent system of humbuggery. The newspapers teem with such advertisements as the above—of parties who will send a formula *free* for certain ailments, "having only in view a desire to benefit humanity," &c. Any person with half an eye, can detect the miserable swindle. In the first place community is not blest with men of that amount of benevolence that will induce them to furnish folks with the means of curing disease, *and pay for their own advertising, too*, all for nothing. Then, any sensible man can discern that the formula given is all *bosh.* No such ingredients as the kind, benevolent widow (?) offers are to be found only in her own laboratory; and of course she expects you to forward the *five dollars*, and procure the medicine (?) of her.

The other "unknown lady" who wrote you the letter, is of course an accomplice of the widow, who doubtless shares her income for writing bushels of just such letters as you received.

As for the other chap with his "Organic Vibrators"—let him vibrate, alone—it will be better for your organs.

With regard to curing your hearing, there is probably no cure for you, unless some mechanical obstruction of the ear exists. Persons of your age are rarely benefitted by a treatment for deafness.

—A frog three feet four inches high, weighing ninety-four pounds, and going ten feet at a leap, is one of the products of Tennessee. Fact: the newspapers say so.—*Advertiser*.

Where is Mark Twain? He wants that frog.

